# Supplementary material for: Content-rich biological network constructed by mining PubMed abstracts
Source: BMC Bioinformatics. 2004 Oct 8;5:147. doi: 10.1186/1471-2105-5-147 (PMC528731; doi:10.1186/1471-2105-5-147)
Supplement: Additional File 5 — The original Chilibot query results of the term "long-term potentiation (LTP)" and 22 other terms, limiting the latest references analyzed to the years 1990, 1995, 2000, and 2004. [file 1471-2105-5-147-S5.bz2 › chilibotAdditionalFile5/ltp2004/html/SYNAPTOTAGMIN.html]

 


**SYNAPTOTAGMIN** (Input: SYNAPTOTAGMIN ) 

---


**Google Searches:** Entire Web  | EDU domain only  | PDF files only

.

**External Links:** OMIM |
LocusLink
| Swissprot
| GeneCards

  
**Maps of SYNAPTOTAGMIN**

Simple
Complete
graph in
radiant
tree
square
layout.

**New Hypothesis !**

**Synonyms** 

- synaptotagmin   [PubMed]

**Synopsis**

- The results suggest the possibility that the phosphorylation of **synaptotagmin** I by CaMKII and PKC contributes to the mechanism s by which these two kinases regulate neurotransmitter release.  J Neurochem, 1999 
  [23]

- Our results show that the levels of synapsin I and **synaptotagmin** I are increased in the hypothyroid rats, which suggests that the genes encoding these proteins are implicated in the action of thyroid hormone on neurotransmitter release.Taken together, the results from this study suggest that thyroid hormone may modulate the probability of neurotransmitter release.  Neuroscience, 2002 
  [20]

- Complexes containing CSP and **synaptotagmin** were immunoprecipitated from rat brain membranes, further suggesting that these proteins interact in vivo.  Biochem J, 2002 
  [18]

- Here we review the role of the synaptic protein interaction synprint sites in the intracellular loop II III L II III of both alpha1B and alpha1A subunits of N type and P Q type calcium channels, which bind to syntaxin, SNAP 25, and **synaptotagmin**.  J Bioenerg Biomembr, 1998 
  [16]

- The similarity between the structures of the **synaptotagmin** I C2A domain and the PLC delta1 C2 domain suggests that the latter binds four calcium ions and acts by a similar mechanism.  Biochemistry, 1998 
  [16]

- Thus, N copine may have a role as a calcium sensor in postsynaptic events, in contrast to the known roles of double C2 domain containing proteins, including **synaptotagmin** I, in presynaptic events.  J Neurochem, 1999 
  [16]

- The modulation of ischemic hippocampal damage by the in vivo knockdown of **synaptotagmin** I or synapsin I suggests that transmitter release from terminals plays an important role in the evolution of ischemic brain damage and therefore the transmitter release strategy by the use of antisense ODNs HVJ liposome complex is reliable for neuroprotective therapies.  Neurosci Res, 2003 
  [16]

- Despite the nearly constant rates of synthesis of **synaptotagmin** I, vamp 2, and synapsin I, we show that the number of SVs in these developing neurons increases,.  J Neurosci, 1997 
  [16]

- There was a down regulation of synaptophysin, **synaptotagmin**, and protein phosphatases PP1alpha and PP1beta mRNAs in AD.  Neurochem Res, 2002 
  [13]

- The results demonstrate that chronic treatment with fluoxetine and tranylcypromine leads to an increased expression of synaptophysin, but decreased expression of **synaptotagmin** and VAMP in the hippocampus and cerebral cortex.  Pharmacogenomics JPharmacogenomics J, 2002 
  [11]

- Neuroscience, 1994 
  [10]

- The eight stranded, Greek key beta sandwich fold of PKCbeta C2 is similar to that of the **synaptotagmin** I type I C2 domain.  Structure, 1998 
  [10]

- Phosphorylation dephosphorylation cycle of **synaptotagmin** can be important for regulation of recyclization of synaptic vesicles.  Biokhimiia, 1996 
  [10]

- They carry the calcium sensor **synaptotagmin**, small GTP binding proteins of the rab3 group or the v SNARE synaptobrevin.  Neurochem Int, 1997 
  [10]

- The conditions used indicate that **synaptotagmin**, as synapsin I, is phosphorylated by calcium calmodulin dependent protein kinase II.  FEBS Lett, 1993 
  [10]
